# Association of dietary pattern with carotid intima media thickness among children with overweight or obesity

**DOI:** 10.1186/s13098-019-0472-4

**Published:** 2019-09-11

**Authors:** Assa Akbari-Sedigh, Golaleh Asghari, Emad Yuzbashian, Pooneh Dehghan, Hossein Imani, Parvin Mirmiran

**Affiliations:** 1 Department of Nutrition, School of Nutritional Science Dietetics, Tehran University of Medical Sciences, P.O. Box: 141664-3931, Tehran, Iran; 2 Nutrition and Endocrine Research Center, Research Institute for Endocrine Sciences, Shahid Beheshti University of Medical Sciences, P.O. Box: 19395-4763, Tehran, Iran; 3 Department of Imaging, Research Development Center, Taleghani Hospital, Shahid Beheshti University of Medical Sciences, Tehran, Iran

**Keywords:** Adolescents, Carotid intima media thickness (cIMT), Children, Dietary pattern, Obese

## Abstract

**Background:**

Since there is no evidence demonstrating the relationship between dietary patterns and subclinical atherosclerosis in children and adolescents, we aimed to examine the association between dietary patterns and carotid intima-media thickness (cIMT) in children and adolescents with overweight and obesity.

**Methods:**

Data were collected on individuals, aged 6–13 years (n = 339) recruited from primary schools with age- and sex-specific body mass index (BMI) Z-score > 1, based on WHO criteria. Dietary intake was assessed using a valid and reliable food frequency questionnaire and dietary patterns were derived by factor analysis. Measurement of cIMT was performed by means of ultrasonography for the wall of common carotid artery.

**Results:**

The mean ± SD age, BMI Z-score and cIMT of study participants were 9.3 ± 1.7 years, 2.5 ± 0.7 and 0.403 ± 0.057 mm, respectively. Three dietary patterns were identified, which accounted for 23.0% of the total variance, including the healthy, the traditional, and the unhealthy patterns. After adjusting for age, sex, pubertal status, smoking exposure, physical activity, body fat percentage, and intake of magnesium and energy, a significant inverse association was observed between the healthy dietary pattern and cIMT (β = − 0.131, P = 0.019), whereas none were found between cIMT and the traditional (β = − 0.004, P = 0.932) and the unhealthy dietary (β = 0.004, P = 0.942) patterns.

**Conclusions:**

Results of the present study indicate that adherence to healthy dietary pattern could prevent increased cIMT in children and adolescents with overweight and obesity. Further cohort design research is required to elucidate the association between dietary patterns and cIMT in children and adolescents.

## Background

Atherosclerosis, the major cause of cardiovascular diseases (CVD), begins in childhood as arterial damage in the intima of large muscular arteries [1, 2]. Evidence showed that excess weight in childhood has a positive relationship with CVD risk factors in adulthood [3]. Hence, the assessment of subclinical markers of atherosclerosis may help follow the progression of atherosclerosis in obese children and adolescents [4]. Measurement of carotid intima-media thickness (cIMT), as a noninvasive method and a surrogate marker for early alterations of atherosclerosis, is an independent predictive factor of CVD [5]. Because children and adolescents with obesity are at higher risk for increased cIMT, determining the most associated risk factors is crucial [6].

Modifiable lifestyle factors, such as diet and physical activity, also play important roles in obesity-related artery damage and dysfunction [7]. There is some evidence on the association of dietary intakes with cIMT in children and adolescents [8–13]. In a study conducted by Giannini et al. [13] cIMT significantly decreased after a 12-month intervention using a Mediterranean diet in pre-pubertal children with hypercholesterolemia. In children with epilepsy, ketogenic diet had no effects on cIMT [8, 9]; however, in another study, this diet increased arterial stiffness [10]. Benefits of hypocaloric low-glycemic index diet on cIMT and stiffness in obese children have been reported by Iannuzzi et al. [11]. Moreover, there was a study aimed to determine the extent to which typical childhood dietary trajectories predict adolescent cardiovascular phenotypes; in this regard, decade-long dietary trajectories did not appear to affect macro- or microvascular structure or stiffness by mid-adolescence, but were associated with resting heart rate, suggesting an early-life window for prevention [12]. All aforementioned studies indicate that dietary intake could influence cIMT and artery structure. However, these studies did not consider dietary patterns based on principal components analysis (PCA), which is a required approach to overcome the interaction and synergistic effects of nutrients and foods. Therefore, dietary patterns as a better choice rather than the assessment of a single or few nutrients or foods in examining the association between diet and chronic diseases, makes utilizing dietary patterns easy for the public in dietary guidelines [14].

Evaluating diet using a multi-dimensional and food-based approach may improve our understanding of which dietary patterns may have associations with cIMT in children and adolescents. Thus, the purpose of the present study was to evaluate the association of dietary patterns with cIMT among children and adolescents with overweight or obesity.

## Method

### Study population

This cross-sectional study was conducted on children and adolescents aged 6 to 13 years, with age- and sex-specific body mass index (BMI) Z-scores ≥ 1 (according to criteria established by the World Health Organization), were recruited from primary schools located in districts of Tehran. The eligibility criteria applied for the current study was: no known medical illnesses such as diabetes, kidney or liver disease (based on physician examination and medical records review), no taken any drug or supplementation, and no on specific diet during the past year. An alphabet list of all eligible students was prepared and then a simple random sampling was generated. Finally, 378 students were invited to the Research Institute for Endocrine Sciences (RIES). We excluded participants with missing data for cIMT (n = 11), dietary intakes (n = 12), and those who over and under-reported total energy intakes (n = 16). Eventually, 339 children remained for final analysis.

Written informed consent was obtained from participants’ parents or legal guardians.

### Dietary assessments

Dietary intakes were collected using a valid and reliable semi-quantitative food frequency questionnaire (FFQ) which was completed via a trained dietitian during a face to face personal interview. Interviewers used photographs of household portions in order to confirm exact food intakes. When children are unable to recall, mothers are asked about the type, quantity, and frequency of meals and snacks during the past year. Four possibilities of frequency, including day, week, month, and year were offered for each food item. Since the Iranian food composition table (FCT) is incomplete, the US Department of Agriculture FCT was used as the Iranian FCT was the alternative for traditional foods not listed in the US Department of Agriculture FCT.

The validity of the FFQ has been reported previously [15]. In brief, over the 1-year interval, 12 dietary recalls (DRs) were collected (1 each month) by the same trained dietitians according to a standardized protocol. Afterwards, the FFQ was collected 1 month after the last recall. An appropriate correlation was observed between dietary patterns derived from the FFQ and the DRs [15].

### Measurements of covariates

We have assessed body composition by a portable bioelectrical impedance analyzer (BIA; Model, GAIA 359 Plus, Co. Cosmed, Italy). Participants stood with soles in contact with the foot electrodes, while holding the hand electrodes. Output data included body weight (kilograms) close 0.1 kg and body fat percentage as calculated by the manufacturer software. In addition, waist circumference (WC) at the umbilicus, using a measuring tape without pressure to body surfaces, and height was measured to the nearest 0.5 cm in a standing position, without shoes, using a measuring tape while the shoulders were in a normal position; and BMI was calculated as weight (kg)/height (m)^2^. Arterial blood pressure was measured twice manually, using a mercury sphygmomanometer with a suitable cuff size for each participant after a 15-min rest. Systolic blood pressure (SBP) was determined by the onset of the tapping sound, and diastolic blood pressure (DBP) was determined at the disappearance of the sound, the second value was considered as the participant’s blood pressure. In addition, a blood sample was drawn into vacutainer tubes from all participants between 7:00 a.m. and 9:00 a.m. after 12–14 h overnight fasting. To determine low density lipoprotein (LDL-C) concentrations, the Friedewald equation was used [16].

Physical activity was assessed using the Modifiable Activity Questionnaire (MAQ) to calculate metabolic equivalent task minutes per week. It is notable that a high reliability (97%) and moderate validity (49%) have been ascertained previously among adolescents for the Persian translated MAQ [17].

Pubertal status was classified according to the definitions of Tanner stages by a well-trained physician. The pubertal developmental stage was categorized into 5 groups based on breast and genital stages (pre-pubertal: boys at genital stage I, girls at breast stage I; pubertal: boys at genital stages II–IV, girls at breast stages II–IV) [18, 19].

### cIMT measurement

In this study, carotid artery ultrasonography was performed by a well-trained technician. Participants were examined in the supine position, the neck slightly extended, and the head turned away to the side; the carotid arteries were interrogated using a high-resolution Samsung ultrasound machine (model UGEO WS80A) with a linear-array transducer operating at a frequency of at least 7 MHz; depth, gain, and focus were adjusted for each participant individually so that the arterial lumen was completely anechoic and in the center of the image. Common cIMT was measured from the longitudinal B-mode images of the distal 1 cm of the far wall of common carotid artery (CCA) between the intimal-luminal and the medial-adventitial interfaces of the carotid artery wall, presented as a double-line density on the ultrasound image. In rare cases where appropriate images of distal CCA could not be obtained, proximal or mid-CCA images were used for cIMT measurement. Measurements were performed using automated edge-tracking software (automated cIMT calculator), which obviated the need to perform manual measurements.

### Statistical analysis

Kolmogorov–Smirnov test and the histogram chart was used to assess the normality of distribution of variables. Normal quantitative variables are expressed as the mean ± standard deviation (SD), skewed and categorical ones as median (25–75 interquartile range) and percentages, respectively.

Food items were classified into 24 food groups based on similar nutrient profiles or their culinary use according to previous study [15] with few modifications to adjust for children and adolescents (Additional file 1: Table S1). After the adjustment of food groups for total energy intake using residual methods, principal component analysis (PCA) with varimax rotation was used to determine major dietary patterns. Factor loadings which represent the correlation coefficients between the different food groups in each dietary pattern identified important food groups contributing to each dietary pattern. A scree plot showed the eigenvalues and the number of factors (dietary patterns) on the x-axis. The point where the slope of the curve is clearly leveling off indicates the number of factors that should be generated by the analysis. Thus, we chose eigenvalue ≥ 1 due to the upward curve. The factor scores for each individual were calculated by summing the frequency of consumption multiplied by factor loadings across all food groups with loadings above 0.2. Dietary pattern scores were categorized into tertiles.

Multivariable linear regression analysis was applied for evaluating the relationship between dietary patterns and cIMT. Confounding variables were age, sex, pubertal status, passive smoking, physical activity, body fat percentage, and magnesium and energy intake. Before conducting multivariable linear models, the interaction terms of dietary pattern scores with age, gender, and BMI categories including overweight subjects (BMI 75th to  < 90th percentiles), obese subjects (BMI 90th to  < 97th percentiles) and super-obese subjects (BMI > 97th percentiles) on cIMT were examined; no interaction was observed between these covariates and dietary patterns. All data analyses were performed using IBM SPSS for Windows (SPSS, Chicago, IL, USA) with the significance level set at P < 0.05 (two-tailed).

Sensitivity analyses were conducted to test the robustness of the results. We further adjusted with the second measurements of SBP and DBP, as well as FPG, LDL-C, total cholesterol, and triglycerides concentrations. Besides, we have also adjusted for BMI and WC instead of body fat percentage in the final model.

### Ethics approval

The protocol of this study was approved by the ethics committee of the Research Institute for Endocrine Sciences, affiliated with the Shahid Beheshti University of Medical Sciences.

## Results

This cross-sectional study included 339 children and adolescents, 17.4% of whom were in Tanner stage I; mean ± SD age of the participants was 9.3 ± 1.7 years; moreover, the average BMI Z-score was 2.55, and the mean ± SD of 0.403 ± 0.057 mm was obtained for cIMT.

Three dietary patterns were extracted, which explained 23.0% contribution for cumulative variance. Factor loadings for each food group are presented in Table 1; the healthy pattern was highly correlated with an intake of fruits and dried fruits, vegetables, fruit juices, low-fat dairy, legumes, and corn. The traditional pattern demonstrated high factor loadings for poultry, red and organ meats, fish and nuts. The unhealthy pattern was highly correlated with fast foods, sugar sweetened beverages, high-fat dairy, sugars, tea and coffee, eggs, French fries, and animal fat.

**Table 1:** Dietary patterns and factor loadings of specific food groups obtained from FFQ (n = 339)

Food groups	Factor 1	Factor 2	Factor 3
Fruits	0.7	–	–
Vegetables	0.6	–	–
Fruit juice	0.4	–	–
Dried fruits	0.4	–	–
Low fat dairy	0.4	–	− 0.2
Legumes	0.2	–	− 0.2
Corn	0.2	–	–
Fast foods	− 0.2	–	0.2
Sugar sweetened beverages	− 0.2	0.2	0.2
Refined grains	− 0.2	− 0.5	–
High fat dairy	− 0.3	–	0.2
Poultry	–	0.6	–
Red and organ meats	–	0.6	–
Fish	–	0.5	–
Nuts	–	0.4	− 0.2
Snacks	–	− 0.3	–
Sugars	–	–	0.4
Tea and coffee	–	–	0.4
Eggs	–	–	0.2
French fries	–	–	0.2
Animal fat	–	–	0.2
Vegetable fat	–	–	− 0.3
Whole grains	–	–	− 0.6
Pickles	–	–	–

Factor loadings > 0.2 represent significant contributions of food groups for the components

*FFQ* food frequency questionnaire

Participants’ characteristics are presented in Table 2 according to the tertiles of extracted dietary patterns; participants with higher scores in healthy dietary pattern spent a lesser time watching TV. In addition, interestingly, participants with higher scores of unhealthy dietary pattern had lower SBP.

**Table 2:** Demographic and clinical characteristics of children and adolescents with overweight and obesity by tertiles of dietary patterns

	Tertiles of dietary patterns			P for trend
	T1	T2	T3	
Healthy pattern				
Age (years)	9.3 ± 1.8	9.4 ± 1.8	9.1 ± 1.6	0.264
Girls (%)	38.9	49.1	55.3	0.013
Pre-puberty (%)	20.4	16.1	15.8	0.366
BMI Z-score	2.6 ± 0.7	2.4 ± 0.6	2.5 ± 0.6	0.513
Body fat (%)	26.8 ± 6.9	26.0 ± 6.7	25.4 ± 6.2	0.215
Systolic BP (mmHg)	100.0 (90.0–110.0)	100.0 (96.5–113.0)	100.0 (95.0–110.0)	0.706
Diastolic BP (mmHg)	60.0 (60.0–70.0)	65.0 (60.0–70.0)	60.0 (60.0–70.0)	0.109
Watching TV (h/day)	3.5 ± 0.95	3.6 ± 0.97	3.3 ± 0.94	0.040
Smoking exposure (%)	36.6	32.9	30.5	0.391
LDL-C (mg/dl)	97.8 ± 25.6	97.0 ± 26.9	97.5 ± 25.4	0.974
cIMT (mm)	0.400 ± 0.053	0.407 ± 0.063	0.401 ± 0.056	0.998
Traditional pattern				
Age (years)	9.5 ± 1.7	9.2 ± 1.6	9.1 ± 1.8	0.221
Girls (%)	46.0	51.3	46.0	1.000
Pre-puberty (%)	15.0	17.7	19.5	0.381
BMI Z-score	2.5 ± 0.7	2.5 ± 0.7	2.5 ± 0.6	0.981
Body fat (%)	25.4 ± 6.4	26.7 ± 5.7	26.1 ± 7.6	0.166
Systolic BP (mmHg)	100.0 (96.5–113.0)	100.0 (99.0–115.0)	101.5 (90.0–110.0)	0.855
Diastolic BP (mmHg)	60.0 (60.0–70.0)	63.0 (60.0–70.0)	61.0 (60.0–70.0)	0.761
Watching TV (h/day)	3.4 ± 1.0	3.5 ± 0.8	3.4 ± 1.0	0.990
Smoke exposure (%)	37.8	26.8	35.4	0.663
LDL-C (mg/dl)	95.8 ± 23.6	94.3 ± 27.3	102.2 ± 26.3	0.659
cIMT (mm)	0.401 ± 0.064	0.403 ± 0.058	0.404 ± 0.049	0.474
Unhealthy pattern				
Age (year)	9.3 ± 1.6	9.2 ± 1.6	9.3 ± 1.8	0.957
Girls (%)	48.2	53.1	42.1	0.355
Pre-puberty (%)	13.4	16.8	21.9	0.090
BMI Z-score	2.5 ± 0.7	2.5 ± 0.7	2.5 ± 0.6	0.940
Body fat (%)	25.9 ± 6.7	26.9 ± 5.7	25.3 ± 7.4	0.852
Systolic BP (mmHg)	103.0 (100.0–115.0)	100.0 (90.0–110.0)	100.0 (93.7–110.0)	0.043
Diastolic BP (mmHg)	65.0 (60.0–70.0)	60.0 (60.0–70.0)	60.0 (60.0–70.0)	0.139
Watching TV (h/day)	3.3 ± 0.9	3.5 ± 0.9	3.5 ± 1.0	0.178
Smoke exposure (%)	23.2	32.9	43.9	0.016
LDL-C (mg/dl)	96.4 ± 26.9	98.8 ± 27.5	97.1 ± 23.5	0.623
cIMT (mm)	0.399 ± 0.056	0.398 ± 0.057	0.411 ± 0.059	0.153

The χ^2^ test was used for categorical variables and linear regression for continuous variables

*BMI* body mass index, *BP* blood pressure, *LDL-C* low-density lipoprotein cholesterol, *cIMT* carotid intima-media thickness

* P-value < 0.05 is significant

Food intakes in the tertiles of three dietary patterns are illustrated in Table 3. Those participants had higher adherence to the healthy dietary pattern had higher intakes of carbohydrate, total fiber, potassium, magnesium, calcium, and lower intakes of fat, monounsaturated fatty acid (MUFA), and sodium. Furthermore, compared to those in the bottom tertiles, participants who were in the top tertiles of the traditional dietary pattern had lower intakes of energy, carbohydrate, polyunsaturated fatty acid (PUFA), and total fiber as well as the higher intakes of protein and saturated fatty acid (SFA), sodium, potassium, magnesium, and calcium. Higher adherence to the unhealthy dietary pattern was accompanied by lower intakes of protein and magnesium and higher intakes of SFA.

**Table 3:** Macronutrient and micronutrient intakes according to the tertiles of extracted dietary patterns in children and adolescents

	Tertiles of dietary patterns			P for trend
	T1	T2	T3	
Healthy pattern				
Energy (kcal)	3032 ± 879	2597 ± 731	2922 ± 969	0.176
Carbohydrate (% energy)	54.4 ± 6.4	55.4 ± 4.4	58.1 ± 5.5	< 0.001
Protein (% energy)	13.3 ± 2.2	13.3 ± 2.1	13.4 ± 2.1	0.697
Fat (% energy)	33.9 ± 6.08	33.4 ± 4.6	31.6 ± 5.4	0.001
Saturated fatty acid (% energy)	10.6 ± 2.7	10.4 ± 2.1	9.7 ± 2.0	0.004
Monounsaturated fatty acid (% energy)	10.8 ± 2.5	10.5 ± 1.8	9.9 ± 2.4	0.008
Polyunsaturated fatty acid (% energy)	7.2 ± 2.1	6.9 ± 1.7	6.7 ± 2.0	0.088
Total fiber (g/1000 kcal)	16.3 ± 6.5	17.2 ± 6.0	18.6 ± 5.0	0.008
Sodium (mg/1000 kcal)	1406 ± 371	1489 ± 495	1356 ± 383	0.047
Potassium (mg/1000 kcal)	1286 ± 220	1449 ± 203	1748 ± 317	< 0.001
Magnesium (mg/1000 kcal)	145 ± 29	152 ± 23	167 ± 25	< 0.001
Calcium (mg/1000 kcal)	453 ± 129	495 ± 114	532 ± 141	< 0.001
Traditional pattern				
Energy (kcal)	3123 ± 869	2624 ± 742	2807 ± 959	< 0.001
Carbohydrate (%energy)	57.5 ± 6.1	56.5 ± 4.4	53.9 ± 5.8	0.003
Protein (%energy)	12.08 ± 1.5	13.2 ± 1.9	14.8 ± 1.9	< 0.001
Fat (%energy)	32.5 ± 6.3	32.6 ± 4.3	33.8 ± 5.5	0.374
Saturated fatty acid (%energy)	9.8 ± 2.4	10.5 ± 2.2	10.4 ± 2.3	0.018
Monounsaturated fatty acid (%energy)	10.5 ± 2.6	9.9 ± 1.6	10.7 ± 2.4	0.360
Polyunsaturated fatty acid (%energy)	7.3 ± 2.2	6.5 ± 1.5	7.0 ± 2.0	0.004
Total fiber (g/1000 kcal)	19.0 ± 7.1	17.6 ± 5.3	15.5 ± 4.5	0.001
Sodium (mg/1000 kcal)	1340 ± 360	1451 ± 496	1459 ± 391	0.020
Potassium (mg/1000 kcal)	1414 ± 354	1524 ± 307	1549 ± 268	0.001
Magnesium (mg/1000 kcal)	145 ± 24	156 ± 23	164 ± 30	< 0.001
Calcium (mg/1000 kcal)	443 ± 118	524 ± 130	513 ± 134	< 0.001
Unhealthy pattern				
Energy (kcal)	2948 ± 713	2637 ± 838	2969 ± 1034	0.200
Carbohydrate (%energy)	56.2 ± 5.7	56.3 ± 5.5	55.5 ± 5.8	0.641
Protein (%energy)	13.9 ± 2.1	13.1 ± 1.9	12.9 ± 2.1	< 0.001
Fat (%energy)	32.2 ± 6.1	32.9 ± 5.1	33.8 ± 5.1	0.072
Saturated fatty acid (%energy)	9.6 ± 9.6	10.2 ± 2.1	10.8 ± 2.4	0.001
Monounsaturated fatty acid (%energy)	10.3 ± 2.5	10.3 ± 2.2	10.4 ± 2.1	0.874
Polyunsaturated fatty acid (%energy)	7.1 ± 2.2	7.0 ± 2.0	6.7 ± 1.7	0.336
Total fiber (g/1000 kcal)	17.7 ± 5.9	18.0 ± 5.2	16.5 ± 6.5	0.463
Sodium (mg/1000 kcal)	1420 ± 485	1405 ± 360	1425 ± 416	0.931
Potassium (mg)/1000 kcal)	1532 ± 330	1473 ± 287	1482 ± 329	0.139
Magnesium (mg/1000 kcal)	168 ± 31	151 ± 20	146 ± 24	< 0.001
Calcium (mg/1000 kcal)	524 ± 150	477 ± 113	479 ± 127	0.003

Linear regression was used for continuous variables

* P-value < 0.05 is significant

The association between dietary patterns and cIMT is shown in Table 4. In the crude model and after controlling for potential confounders, we found a statistically significant inverse association between healthy dietary pattern and cIMT (β = − 0.131, P = 0.019). Although, there was no significant association of traditional and unhealthy dietary patterns with cIMT.

**Table 4:** Beta coefficient of the dietary patterns and carotid intima-media thickness in children and adolescents with overweight or obesity

	Carotid intima-media thickness	
	Beta coefficient	P value
Healthy pattern		
Model 1	− 0.121	0.032
Model 2	− 0.131	0.019
Traditional pattern		
Model 1	− 0.034	0.547
Model 2	− 0.004	0.932
Unhealthy pattern		
Model 1	− 0.019	0.740
Model 2	0.004	0.942

Model 1: crude

Model 2: adjusted for age, sex, puberty status, smoke exposure, leisure physical activity, magnesium intake, energy intake, percent body fat

* P-value < 0.05 is significant

In sensitivity analyses, results were not substantially changed when we further adjusted with SBP and DBP, and total cholesterol, triglycerides, and LDL-C concentration as well as FPS. Furthermore, neither BMI nor WC altered final findings when they were adjusted for, instead of body fat percentage measurement in multivariable regression.

## Discussion

The aim of our study was to investigate the association between dietary pattern and cIMT in children and adolescents with overweight and obesity. Based on three major extracted dietary patterns, we found the healthy dietary pattern to be inversely associated with cIMT after adjustment for potential confounders. However, there was no significant association of cIMT with traditional and unhealthy patterns.

The exploratory dietary pattern has become a popular approach in nutritional epidemiology [20]. Factor analysis, as a posteriori method, identified dietary pattern through PCA which could be used for planning behavioral interventions based on dietary behaviors [21]. A number of studies have investigated the association between dietary pattern with cIMT and endothelial dysfunction among adults [21–26]. Similar to our unhealthy pattern, a dietary pattern characterized by high intake of processed meats, fried potatoes, and desserts, had no significant association with cIMT in the Multi-Ethnic Study of Atherosclerosis (MESA) population [21]. Likewise, Liese et al. [23] observed that adhering to a dietary pattern characterized by higher intakes of low-fiber bread and cereal, red and processed meat, and sweetened beverages and lower intakes of rice, pasta, and poultry, caused a 13 µm increase in cIMT during a 5-year follow-up. The Young Finns Study among children and adolescents with a 21-year follow up, showed that a traditional dietary pattern (rye, potatoes, milk, butter, sausages, and coffee) was positively associated with cIMT in men, an association not observed in our study [24]; one possible explanation for this inconsistency is that nuts and fish, two food groups in our traditional pattern, could have counteracted the detrimental effects of the traditional diet. Contrary to our findings, there was a significant correlation between unhealthy dietary pattern and cIMT although none was found with the healthy dietary pattern in older adults [25]. However, when participants were categorized by the highest and lowest intakes of fruits and vegetables, the high intake group obtained significantly lower cIMT values in Da Silva et al. study [25]. Identifying dietary patterns and their relationships with health promotion could be beneficial to elucidate escalating indicators of atherosclerosis.

Previous studies have reported significant relationships between cIMT and other CVD risk factors, including obesity, metabolic syndrome, excess visceral adipose tissue, and blood pressure in childhood which could be a result of undesirable eating behaviors [27–31]. A systematic review study indicated that adiposity measured by BMI, body fat percentage, weight status and WC had a positive correlation with cIMT in adolescents, but not in younger children [32]. Some studies have illustrated the associations between food patterns and CVD risk factors among children [33–35]. A systematic review reported that dietary patterns which had energy dense, high fat and low fiber foods were related to increased body weight in children and adolescents [33]. A nationwide cross-sectional study showed that a dietary pattern characterized by high cheese and red processed meat increased blood pressure in 10–12 year children probably by increasing their sodium intake [34]. Furthermore, a longitudinal study demonstrated that metabolic syndrome is predictive of cIMT in children, and the improvement of metabolic status reduced the atherosclerosis process at follow-up [35]. Accordingly, it is plausible that changes in health-oriented dietary patterns and the improved CVD risks may reduce subclinical atherosclerosis.

Data on observed associations in literature can be related to the known effects of nutrients in dietary patterns. Participants on the healthy dietary pattern had higher intakes of calcium, magnesium, potassium, and fiber. A previous study reported that the low concentration of magnesium had an association with endothelial dysfunction, which might accelerate the progression of atherosclerosis and increase of cIMT [36]. According to the study conducted by Buil-Cosiales, the higher intake of fiber was inversely associated with cIMT and carotid atherosclerosis [13]. The protective effect of fiber might be due to its blood pressure and cholesterol lowering effect [37, 38]. In addition, participants who had higher scores in our healthy dietary pattern consumed less amounts of high-fat dairy products. In a cross-sectional study, it was shown that the consumption of 100 g/day low-fat dairy could decrease 0.005 mm of cIMT, indicating, dairy products can play role in subclinical atherosclerosis [39]. Moreover, we found that the unhealthy pattern had lower SBP as an unexpected result which could be related to our cross-sectional design and obtain an exploratory association. Furthermore, blood pressure was considered to be an additional potential confounding factor and was adjusted in multivariable regression. Although trend of blood pressure significantly decreased according to unhealthy pattern, it was not clinically important.

Our results also revealed that the participants who adhered to the healthy dietary pattern had a 0.131 mm decrease in cIMT. There is some evidence indicating that the cut points of carotid wall thickness is predictive of incident clinical stroke or myocardial infarction [40]. An increase in the maximum internal wall thickness, coronary disease could increase by 36% in adults; however, the magnitude of cIMT in association with CVD risk have never been reported in adolescents [41, 42]. Measuring cIMT in children and adolescents with obesity has been recommended by the Association for European Pediatric Cardiology (AEPC), as it simply can identify early vascular alterations and, thus, prevents myocardial infarction and stroke [43]. Early precursors of vascular changes—subclinical atherosclerosis—warrant special attention as this process can be stabilized or even reversed if treated in time. Sonographic intima media thickness measurement of the carotid artery is considered a valid surrogate marker for cardiovascular risk that allows the assessment of atherosclerotic changes at a very early stage. It can easily to be applied due to its non-invasive character.

It is noteworthy that the dietary pattern approach, especially when used through exploratory factor analysis, is strongly dependent on cultural and behavioral differences within the population and, therefore, is not generalizable to other populations. In the “Iranian traditional” and “unhealthy” dietary patterns, unhealthy food groups including fast foods, sugar sweetened beverages, high fat dairy, and animal fat might be highly loaded; however, in our study they had low factor loadings and the presence of some healthy foods like eggs, fish and nuts were also loaded in these dietary patterns. Hence, these healthy foods could interact with other foods in the pattern and compensated for the harmful effects of unhealthy foods in traditional and unhealthy patterns. This seems why, we observed no significant relationship between the traditional and unhealthy patterns with cIMT. However, even if a pattern has been shown to be associated with CVD, the observed pattern cannot been justified to be the most healthy or the most detrimental of all diets [24, 44]. The most plausible justification is that cIMT changes occur over a long time and the effect of unhealthy dietary pattern in young populations will be more pronounced with aging.

Regarding the strengths of our study, it is the first study to examine the association between dietary pattern and cIMT in children and adolescents with overweight and obesity. Moreover, BIA measurements allowed us to control for body fat percentage not just for BMI. This study also has its limitations that should be taken into account. The cross-sectional design could not elucidate whether the thickness of arteries gradually increased during childhood; neither did it not allow us to interpret causal relationships. Although we considered some confounding factors, it is plausible that there were unknown or unmeasured confounders that could not be included in our results. Furthermore, the sample size in our study was not enough to have stratified analysis based on obesity phenotypes and conduct subgroup analysis including healthy obese, unhealthy obese, and obese with metabolic syndrome. Lastly, using an FFQ cannot estimate the ‘actual’ intake of participants.

## Conclusion

In summary, results indicate that the healthy dietary pattern had an inverse association with cIMT. Considering that food behaviors could be identified and controlled in childhood and not eventually affect adult CVD health, we suggest that adhering to healthy dietary patterns as the optimum lifestyle could prevent vascular aging at early stages from childhood. Further research with cohort designs is required to elucidate the association between dietary patterns and cIMT in children and adolescents.

## Supplementary information

**Additional file 1: Table S1.** Food items and food groups.

## Data Availability

The datasets generated and/or analysed during the current study are not publicly available due institution’s policy but are available from the corresponding author on reasonable request.

## Abbreviations

CVD: cardiovascular diseases
cIMT: carotid intima-media thickness
BMI: body mass index
FFQ: food frequency questionnaire
FCT: food composition table
DRs: dietary recalls
BIA: bioelectrical impedance analyzer
WC: waist circumference
SBP: systolic blood pressure
DBP: diastolic blood pressure
LDL-C: low density lipoprotein
MAQ: modifiable activity questionnaire
CCA: common carotid artery
SD: standard deviation
PCA: principal component analysis
MUFA: monounsaturated fatty acid
PUFA: polyunsaturated fatty acid
SFA: saturated fatty acid
MESA: Multi-Ethnic Study of Atherosclerosis
