# Supplementary material for: Association of dietary pattern with carotid intima media thickness among children with overweight or obesity
Source: Diabetol Metab Syndr. 2019 Sep 11;11:77. doi: 10.1186/s13098-019-0472-4 (PMC6740019; doi:10.1186/s13098-019-0472-4)
Supplement: Supplementary file 1 — Additional file 1: Table S1. Food items and food groups. [file 13098_2019_472_MOESM1_ESM.docx]

Table S1. Food items and food groups.

| Food groups | Food items |
| --- | --- |
| Fruits | Orange, tangerine, lemon, lime, grapefruit, banana, apple, pear, strawberry and other berries, peach, cherries, ﬁg, melon, watermelon and Persian melon, cantaloupe, kiwi, apricots, nectarine, mulberry, plums, persimmons, pomegranates, date |
| Fruit juice | Natural juices of apple, orange, and cantaloupe. |
| Dried fruit | Dried apricots, berries, and figs |
| Vegetables | Cabbage, cauliﬂower, kale, carrots, tomatoes, spinach, lettuce, cucumber, eggplant, celery, green peas, green beans, green pepper, turnip, squash, zucchini, mushrooms, onions, garlic, and green leafy vegetables |
| Legumes | Beans, peas, lima beans, broad beans, lentils, soy bean |
| Nuts | Almonds, pistachios, walnuts, hazelnuts, peanuts, roasted seeds |
| Egg | Eggs |
| Red and organ meats | Beef, lamb, ground meat, organ meat (brain, liver, kidney, heart, tongue, feet, and head) |
| Fish | Canned tuna ﬁsh, every kind of ﬁsh, shrimp |
| Poultry | Chicken |
| Whole grains | All whole and dark breads (Barbari, Sangak, and Taftoon), barley, bulgur |
| Refined grains | Lavash, baguettes, noodles, pasta, rice, toasted bread, sweet bread, white flour |
| High-fat dairy | High-fat milk and yoghurt, yoghurt drink (doogh), chocolate milk, concentrated and creamy yoghurt, ice cream, cream cheese, other cheeses, kashk |
| Low-fat dairy | Low- fat milk, yogurt and cheese |
| Fast foods | Hamburger, sausages, pizza |
| French fries | French fries |
| Snacks | Potato chips, corn puffs, crackers, biscuits, popcorn |
| Sugars | Sugar, cube sugar, candies, Iranian confectioneries (gaz, Sohan, noghl), jam, jelly, honey, chocolates, cookies, cakes, confections, caramels |
| Sugar-sweetened beverages | All soft and sweet drinks, beer (non-alcoholic), syrup |
| Vegetable fat | Vegetable oils, hydrogenated vegetable oils, mayonnaise, margarine, olive oil, olive |
| Animal fat | Animal fats, butter, cream |
| Corn | Corn |
| Pickles | Pickles |
| Tea and coffee | Tea, coffee |
